# Phenotypic Features of Isolated Essential Tremor, Essential Tremor Plus, and Essential Tremor-Parkinson’s Disease in a Movement Disorders Clinic

**DOI:** 10.5334/tohm.581

**Published:** 2021-03-29

**Authors:** Steven T. Bellows, Joseph Jankovic

**Affiliations:** 1 Parkinson’s Disease Center and Movement Disorders Clinic, Department of Neurology, Baylor College of Medicine, Houston, TX

**Keywords:** essential, tremor, plus, Parkinson

## Abstract

**Background:**

Patients with essential tremor were initially considered to have isolated tremor, but additional motor and non-motor features have been increasingly recognized. The term “essential tremor plus” was adopted by the Task Force on Tremor of the International Parkinson and Movement Disorder Society to describe essential tremor patients with additional neurologic signs.

**Objectives:**

To characterize essential tremor patients and their phenotypes in a movement disorders clinic population in the context of the new tremor classification.

**Methods:**

Demographic, clinical, historical, treatment, and diagnostic data were retrospectively collected on 300 patients diagnosed by movement disorder experts with essential tremor. Patients were classified as having essential tremor, essential tremor plus, or essential tremor-Parkinson’s disease combination, and features between these groups were compared.

**Results:**

Of the 300 patients, 20.7% were classified as isolated essential tremor, 53.3% as essential tremor plus, and 26.0% as essential tremor-Parkinson’s disease. There was no significant difference in the duration of tremor symptoms. Essential tremor plus patients were more likely to have dystonia, tandem gait abnormalities, head tremor and greater tremor severity. Essential tremor-Parkinson’s disease patients were more likely to have RBD symptoms. There was no significant difference in cognitive impairment between essential tremor plus and essential tremor-Parkinson’s disease patients.

**Conclusions:**

Additional motor and non-motor features, including parkinsonism, are common in patients with essential tremor. Further studies are needed to clarify essential tremor phenotypes and to provide insights into possible subtypes.

**Highlights:**

300 patients with essential tremor from a movement disorders clinic were re-classified based on the Movement Disorder Society Consensus Statement on the Classification of Tremors. Additional motor and non-motor features, including parkinsonism, were common, and only 20.7% of patients remained classified as isolated essential tremor.

## Introduction

Since its original description more than a hundred years ago, criteria used to diagnose essential tremor (ET) has varied in different reports and studies. In order to clarify diagnostic criteria, the Movement Disorder Society Consensus Statement on the Classification of Tremors in 2018 adopted a two-axis tremor clarification scheme, consistent of clinical characteristics (Axis 1) and etiology (Axis 2) [1]. Essential tremor was defined as an Axis 1 tremor syndrome with “isolated tremor syndrome of bilateral upper limb action tremor” of “at least 3 years’ duration” in the “absence of other neurological signs, such as dystonia, ataxia, or parkinsonism”. “Essential tremor plus” was adopted to describe tremor with the characteristics of ET as well as “additional neurological signs of uncertain significance” [1], in order to accommodate the additional movement disorders and non-motor symptoms increasingly described in ET patients [2,3,4,5,6,7,8].

These Axis 1 definitions, based on clinical features, and the division into isolated ET and ET plus have been challenged and have created controversy within the field [4,9,10,11,12]. Some studies have found that a majority of patients with a prior diagnosis of ET require reclassification from ET to ET plus [9,10]. Concerns have been raised that ET plus may simply reflect a more advanced stage of the disease, such that some ET patients may eventually “convert” from isolated ET into ET plus over time [11,13]. Given the recently introduced definitions and categories, we aimed to examine the phenotypes of patients previously diagnosed with ET in our movement disorders clinic population in order to characterize the patients in the context of the new tremor classification.

## Methods

This study was a retrospective chart review of patients seen at the Parkinson’s Disease Center and Movement Disorders Clinic at Baylor College of Medicine, Houston, Texas. The study protocol was reviewed and approved by the Institutional Review Board at Baylor College of Medicine. As data was gathered by retrospective chart review, a waiver of informed consent was approved by the same Institutional Review Board. A database of patients who were evaluated in our clinic between May 1, 2016 and May 1, 2020 and carried an ICD10 diagnosis of ET (G25.0) was generated. These patients were randomized through Microsoft® Excel® by assigning each patient a random number with the *rand* function and sorting the patients sequentially by these random number assignments. Randomized patients were then reviewed sequentially and were excluded if they had not been seen in clinic since September 1, 2018, a diagnosis of ET was not confirmed by chart review, or if documentation of their initial clinic visit was missing. In order to reflect a “real-world” experience in a movement disorders clinic we did not exclude patients who had other diagnoses besides ET. The first 300 patients who met these criteria were included for this data analysis.

Clinical, phenomenologic, demographic, family history, and therapeutic data were collected and entered into the database. Historical data collected included family history of tremor, PD, and dystonia, and subjective symptoms of imbalance by patient report. Data based on examination included areas affected by tremor (such as head, voice, tongue, arm, legs, and trunk) and types of tremors seen (including postural, kinetic, rest, and isometric tremors). Some patients were rated using The Essential Tremor Rating Assessment Scale (TETRAS) [14], which includes an objective performance subscore for tremor severity and Activities of Daily Living (ADL) subscore assessing the tremor’s impact on daily function. The presence of additional motor features was noted, including dystonia, parkinsonism, ataxia, tandem gait abnormalities, and other gait abnormalities. Data regarding non-motor features as noted in clinical documentation were collected, including mood, cognitive, and REM Behavior Disorder (RBD), as well as objective cognitive deficits and features of neuropathy on physical examination. Cognitive impairment was defined as a score of 25 or less on the Montreal Cognitive Assessment (MoCA) or a diagnosis of mild cognitive impairment or dementia by formal neuropsychological testing. DaTscan SPECT imaging results were also included when available. Patients were characterized as “ET plus” if they had cognitive impairment determined by a MoCA examination or neuropsychological testing, tandem gait abnormalities, ataxia, dystonia, rest tremor, or other parkinsonian features. The consensus statement classifies patients with “questionable dystonia” as ET plus, while classifying patients with dystonia as a different combined tremor syndrome [1]. While we understand the rationale of this division “for the purpose of facilitating a search for specific etiologies”, from a phenomenological standpoint it is difficult to justify differentiation between “questionable dystonic posturing” and “dystonia” [15,16]. Hence, we included both in the ET plus category. If patients with ET also fulfilled the diagnostic criteria for Parkinson’s disease (PD), as defined by the UK Parkinson’s Disease Society Brain Bank’s Clinical Criteria [17], they were categorized as ET-PD.

The data was entered into a relational database and descriptive statistics were used to determine the prevalence of the various items and features. Further statistical analysis between groups of data was conducted: Chi-squared test was used to compare categorical data, and Fisher’s exact test was used in data with low frequencies. The Shapiro-Wilk test was used to assess for normality of continuous data: normally distributed data was compared between two groups using Student’s t-test, while non-parametric data between two groups was compared using the Mann-Whitney U test. Continuous data with three groups was evaluated first with omnibus one-way ANOVA or the Kruskal-Wallis test, followed by post-hoc Student’s t-test or Mann-Whitney U tests with Bonferroni correction, depending on if the data was normally distributed.

## Results

**Figure 1:**
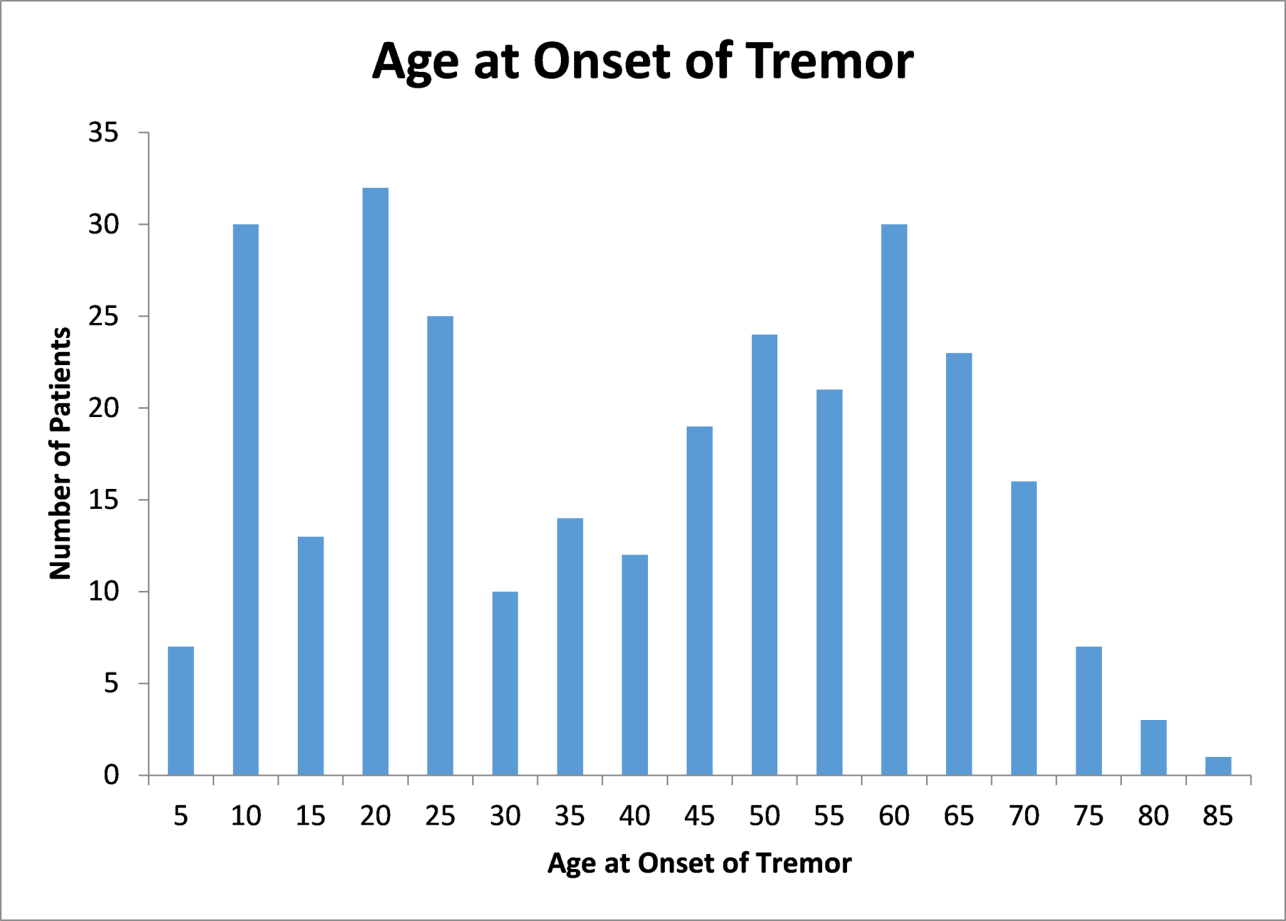
**Age at Onset of Tremor Histogram.** A bimodal distribution is visible in the 2^nd^ and 6^th^ decades of life.

Our study consisted of 300 patients carrying a diagnosis of ET. Using the criteria detailed above, 62 patients (20.7%) were classified as isolated ET, 160 patients (53.3%) as ET plus, and 78 (26.0%) as ET-PD. The prevalence of associated features that resulted in the classification of 160 patients as ET plus is summarized in ***Table 4***. Demographic and historical features are summarized in ***Table 1***. There was a slight (52.3%) male preponderance, and there was a significantly higher percentage of men among ET-PD versus ET plus patients (65.4% vs 43.8%, p = 0.0017). The mean age of our patient population was 66.1 years (range 18–93, SD 13.6), and mean age of isolated ET patients was significantly younger than ET plus patients (60.5 versus 68.1 years, p = 0.00056). The mean age at onset of tremor among all patients was 38.3 years (range 4–81, SD 21.0) with a bimodal distribution suggesting peaks in the 2^nd^ and 6^th^ decades of life, as demonstrated by the histogram in ***Figure 1***. Age at onset of tremor and tremor duration were not significantly different between the three cohorts.

**Table 1:** Demographic and historical features.


	OVERALL	ET	ET PLUS	ET-PD	P (ALL)	ADJ. P (ET VS ET PLUS)	ADJ. P (ET VS ET-PD)	ADJ. P (ET PLUS VS ET-PD)

**Tremor Classification**								

Number of patients (%)	300 (100%)	62 (20.7%)	160 (53.3%)	78 (26.0%)				

**Gender**								

Male	157 (52.3%)	36 (58.1%)	70 (43.8%)	51 (65.4%)	**0.0044**	0.055	0.38	**0.0017**

Female	143 (47.7%)	26 (41.9%)	90 (56.3%)	27 (34.5%)				

**Age, years**								

Mean age (SD)	66.1 (13.6)	60.5 (15.7)	68.1 (13.6)	66.3 (10.2)	**0.00095**	**0.00056**	0.1679	0.0215

Median age	67.0	65.5	70	65				

**Age at Tremor Onset, years**								

Mean age at onset (SD)	38.3 (21.0)	34.3 (19.0)	40.5 (22.0)	36.9 (19.9)	0.13	0.053	0.38	0.29

Median age at onset	41	33	45					

**Tremor duration, years**								

Mean duration (SD)	28.1 (18.8)	26.0 (18.2)	28.1 (19.1)	29.6 (18.9)	0.59	0.47	0.29	0.67

Median duration	23	22	22	25				

**Family History**								

Tremor	215 (71.7%)	40 (64.5%)	123 (76.9%)	52 (66.7%)	0.097	0.061	0.79	0.94

PD	58 (19.3%)	16 (25.8%)	23 (14.4%)	19 (24.4%)	0.066	0.045	0.84	0.058

Dystonia	8 (2.7%)	2 (3.2%)	6 (3.8%)	0 (0.0%)	0.2675	1	0.1943	0.1814

**Clinical Features by History**								

Balance disturbance	82 (27.3%)	10 (16.1%)	44 (27.5%)	28 (35.9%)	**0.033**	0.076	**0.009**	0.19

Depression	89 (29.7%)	16 (25.8%)	49 (30.6%)	24 (30.8%)	0.76	0.48	0.52	0.98

Anxiety	61 (20.3%)	5 (8.1%)	32 (20.0%)	24 (30.8%)	**0.004**	0.03	**0.001**	0.066

RBD symptoms	17 (5.7%)	1 (1.6%)	4 (2.5%)	12 (15.4%)	**0.0003**	1	**0.005**	**0.0002**


Adj. p = adjusted p (statistically significant cutoff of p < 0.017 by Bonferroni correction). SD = standard deviation, PD = Parkinson’s disease, RBD = rapid-eye movement behavior disorder, ET = essential tremor, ET plus = essential tremor plus, ET-PD = essential tremor-Parkinson’s disease.

A majority of patients (71.7%) had a family history of tremor, but there was no significant difference between isolated ET, ET plus, and ET-PD patients. ET-PD patients had a significantly higher prevalence of RBD symptoms compared to isolated ET (15.4% vs 1.6%, p = 0.005) and ET plus (15.4 % vs 2.5%, p = 0.0002). ET-PD patients also had a significantly higher prevalence of anxiety compared to ET patients (30.8% vs 8.1%, p = 0.001).

Features based on examination among the three cohorts are summarized in ***Table 2***. Head tremor was significantly more prevalent in ET plus versus isolated ET (59.4% vs 32.3%, p = 0.00029) and ET-PD (59.4% vs 33.3%, p = 0.00016). Voice tremor was significantly more prevalent in ET plus versus ET-PD (31.9% vs 10.3%, p = 0.00029). Among the entire cohort, 75 patients (25.0%) had dystonia, with the following anatomical distribution: cervical dystonia in 52 (69.3%), limb dystonia in 31 (41.3%), laryngeal dystonia in 7 (9.3%), and blepharospasm in 3 (4.0%). Dystonia (42.5% vs 9.0%, p < 0.0001) and tandem gait abnormalities (38.8% vs 9.0%, p < 0.0001) were significantly more prevalent in ET plus versus ET-PD patients. There was no significant difference in the prevalence of cognitive impairment between ET plus and ET-PD patients (16.3% vs 24.4%, p = 0.134).

**Table 2:** Examination features.


	OVERALL	ET	ET PLUS	ET-PD	P (ALL)	ADJ. P (ET VS ET PLUS)	ADJ. P (ET VS ET-PD)	ADJ. P (ET PLUS VS ET-PD)

**Examination features**								

**Rest tremor**	130 (43.3%)	n/a	64 (40.0%)	66 (84.6%)	n/a	n/a	n/a	<0.0001

**Postural tremor**	294 (98.0%)	62 (100.0%)	157 (98.1%)	75 (96.2%)	0.35	0.56	0.25	0.40

**Kinetic tremor**	290 (96.7%)	62 (100.0%)	156 (97.5%)	72 (92.3%)	**0.033**	0.578	0.034	0.084

**Isometric tremor**	90 (30.0%)	25 (40.3%)	46 (28.8%)	19 (24.4%)	0.11	0.097	0.043	0.48

**Body areas involved**								

**Head**	141 (47.0%)	20 (32.3%)	95 (59.4%)	26 (33.3%)	**<0.0001**	**0.00029**	0.89	**0.00016**

**Voice**	74 (24.7%)	15 (24.2%)	51 (31.9%)	8 (10.3%)	**0.0014**	0.26	0.027	**0.00029**

**Tongue**	41 (13.7%)	7 (11.3%)	27 (16.9%)	7 (9.0%)	0.21	0.30	0.65	0.10

**Upper limbs**	300 (100.0%)	62 (100.0%)	160 (100.0%)	78 (100.0%)	n/a	n/a	n/a	n/a

**Lower limbs**	70 (23.3%)	13 (21.0%)	43 (26.9%)	22 (28.2%)	0.91	0.97	0.71	0.68

**Trunk**	51 (17.0%)	11 (17.7%)	28 (17.5%)	12 (15.4%)	0.91	0.97	0.71	0.68

**Associated features**								

**Parkinsonism**	117 (39.0%)	n/a	39 (24.4%)	78 (100.0%)	n/a	n/a	n/a	**<0.0001**

**Dystonia**	75 (25.0%)	n/a	68 (42.5%)	7 (9.0%)	n/a	n/a	n/a	**<0.0001**

**Tandem gait abnormality**	69 (23.0%)	n/a	62 (38.8%)	7 (9.0%)	n/a	n/a	n/a	**<0.0001**

**Ataxia**	8 (2.7%)	n/a	6 (3.8%)	2 (2.6%)	n/a	n/a	n/a	0.63

**Cognitive impairment**	45 (15.0%)	n/a	26 (16.3%)	19 (24.4%)	n/a	n/a	n/a	0.134

**Neuropathy**	35 (11.7%)	3 (4.8%)	24 (15.0%)	8 (10.3%)	0.10	0.038	0.35	0.31

**TETRAS evaluations**								

**# patients**	88 (29.3%)							

**Mean performance subscore (SD)**	23.4 (6.75)	19.7 (5.2)	24.7 (6.5)	21.8 (7.9)	**0.015**	**0.0047**	0.39	0.15

**Mean ADL subscore (SD)**	21.2 (10.24)	17.9 (8.4)	24.0 (9.9)	12.9 (8.4)	**0.00036**	0.022	0.088	**0.00037**

**Average total score (SD)**	44.8 (15.47)	37.8 (12.6)	49.4 (15.0)	33.6 (12.0)	**0.00022**	**0.00062**	0.36	**0.0005**


Adj. p = adjusted p (statistically significant cutoff of p < 0.017 by Bonferroni correction). SD = standard deviation, ET = essential tremor, ET plus = essential tremor plus, ET-PD = essential tremor-Parkinson’s disease. TETRAS = The Essential Tremor Rating Assessment Scale, ADL = activities of daily living. Cognitive Impairment included MCI or dementia by neuropsychological testing, or MoCA score of ≤ 25. Some TETRAS scores had only ADL or performance subscores available, and were not included in the total score average.

TETRAS scores were available in 88 (29.3%) patients (***Table 2***). Total scores were significantly higher in ET plus patients compared to both isolated ET (49.4 vs 37.8, p = 0.00022) and ET-PD patients (49.4 vs 33.6, p = 0.0005). Performance subscores were significantly higher in ET plus versus isolated ET patients (24.7 vs 19.7, p = 0.0047), while ADL subscores were significantly higher in ET plus versus ET-PD patients (24.0 vs 12.9, p = 0.00037).

Treatment history is summarized in ***Table 3***. ET plus patients had significantly more current medications than patients with isolated ET (1.8 vs 1.4, p = 0.013), while ET-PD had significantly more current medications than ET-plus patients (2.5 vs 1.4, p = 0.0005). ET-PD patients had significantly higher totals of prior medication than isolated ET (4.7 vs 3.1, p < 0.0001) and ET-plus patients (4.7 vs 3.6, p < 0.0001). ET-PD patients were significantly more likely to have at least a partial response to medications than ET plus patients (83.3% vs 65.5%, p = 0.005). BoNT injections were used significantly less in ET-PD than in isolated ET (20.5% vs 41.9%, p = 0.006) or ET plus (20.5% vs 50.6%, p < 0.0001), despite no significant difference in BoNT efficacy among patients who received the treatment. DBS was placed in 38 of all 300 patients with no significant difference between the three cohorts. DBS was effective in 35 of these 38 patients.

**Table 3:** Treatment history.


	OVERALL	ET	ET PLUS	ET-PD	P (ALL)	ADJ. P (ET VS ET PLUS)	ADJ. P (ET VS ET-PD)	ADJ. P (ET PLUS VS ET-PD)

**Medication History**								

**Average # of current oral medications (SD)**	1.92 (1.2)	1.4 (1.0)	1.8 (1.1)	2.5 (1.3)	**<0.0001**	**0.013**	**<0.0001**	**0.00046**

**Median # of current oral medications**	2	1	2	2				

**Average # of prior oral medications (SD)**	3.81 (1.9)	3.1 (1.5)	3.6 (1.8)	4.7 (2.0)	**<0.0001**	0.058	**<0.0001**	**<0.0001**

**Median # of prior oral medications**	4	3	4	5				

**At least partially responsive**	213 (71.0%)	43 (69.4%)	105 (65.5%)	65 (83.3%)	**0.018**	0.60	0.050	**0.005**

**Improved tremor with alcohol**	79 (26.3%)	16 (25.8%)	44 (27.5%)	19 (24.4%)	0.87	0.80	0.84	0.61

**BoNT Injections**								

**Received BoNT injections**	123 (41.0%)	26 (41.9%)	81 (50.6%)	16 (20.5%)	**<0.0001**	0.25	**0.006**	**<0.0001**

**BoNT effective**	91/123 (74.0%)	21 (80.8%)	59 (72.8%)	11 (68.8%)	0.64	0.42	0.46	0.76

**DBS**								

**Underwent DBS placement**	38 (12.7%)	11 (17.7%)	17 (10.6%)	10 (12.8%)	0.36	0.15	0.42	0.62

**DBS effective**	35/38 (92.1%)	11 (100.0%)	15 (88.2%)	9 (90.0%)	0.601	0.51	0.48	1


Adj. p = adjusted p (statistically significant cutoff of p < 0.017 by Bonferroni correction). ET = essential tremor, ET plus = essential tremor plus, ET-PD = essential tremor-Parkinson’s disease, SD = standard deviation, BoNT = botulinum toxin, DBS = deep brain stimulation.

**Table 4:** Essential tremor plus associated features.


FEATURE	NUMBER OF PATIENTS (%), TOTAL N = 160

Rest tremor	64 (40.0%)

Other parkinsonian features	39 (24.4%)

Dystonia	68 (42.5%)

Ataxia	6 (3.8%)

Abnormal tandem gait	62 (38.8%)

Cognitive impairment	26 (16.3%)


DaTscans were performed in 71 patients (23.7%), and 27 of these 71 DaTscans (38.0%) were positive. All 27 of these patients were classified as ET-PD. Among patients with positive DaTscans, the mean latency between onset of tremor and onset of parkinsonian features was 19.2 years, further supporting the co-existence of two disorders, ET and PD. DaTscans were interpreted as “negative” in 3 ET-PD patients. Two of these patients developed worsening clinical parkinsonism after their DaTscan, prompting the diagnosis of PD, and one patient’s DaTscan showed asymmetrically reduced tracer uptake consistent with his examination, suggesting the diagnosis of early PD.

## Discussion

The primary aim of our study was to describe a well-characterized population of patients with ET, diagnosed by movement disorders experts, to determine the prevalence of ET plus in the context of new definitions proposed in the Consensus Statement [1], and to compare features between isolated ET, ET plus, and ET-PD patients. Our 300 patients with clinically defined ET represent one of the largest reported series.

Using the Consensus Statement definition [1], a majority of our patients (72.1%, excluding ET-PD) were classified as ET plus. This is consistent with other recently published studies [9,10,18]. Similar to our prior report, involving 350 patients with ET [19], and other studies [20], the current series again demonstrated a bimodal age-at-onset distribution (***Figure 1***). One study has proposed a trimodal age-at-onset distribution based on mathematical modeling of their cohort of 252 patients, but this observation has not been replicated in other studies [21]. Some have suggested that ET plus may merely represent more advanced stages of ET, but in our population of patients the duration of tremor was not significantly different between isolated ET and ET plus patients. ET plus patients were significantly older than isolated ET patients (mean age 68.1 vs 60.5 years, p = 0.00056); impaired tandem gait (p < 0.0001) and abnormal MoCA scores (Pearson’s r = 0.240, p = 0.016) were also associated with older age.

Although genetics clearly plays an important role in the pathogenesis of ET, family history, noted in 71.7% of our patients, was not included in the diagnostic criteria for ET proposed in the Consensus Statement [1]. There was no significant difference in the prevalence of a family history of tremor between isolated ET, ET plus, and ET-PD patients. Family history of tremor has been previously associated particularly with a younger age at onset of tremor [22], and this association was statistically significant in our study as well (mean 35.7 vs 45.3 years, p = 0.0008). Although many genetic markers have been implicated in ET, no causative genes for ET have been identified [23].

Another common feature of ET, alcohol responsiveness, was “not consistent enough to be included in the definition of ET” in the Consensus Statement [1]. Among our patients in whom an alcohol response could be determined, a majority of patients (79 of 129, 61.2%) noted improvement in their tremor with alcohol, with no significant difference among isolated ET, ET plus, and ET-PD patients. Prior studies have reported similar rates of tremor improvement with alcohol in 1/2 to 2/3 of patients [24,25]. Although alcohol typically improves ET, it should not be recommended as a treatment as it may lead to alcohol abuse [26,27].

While TETRAS scores were available in only 88 patients, ET plus patients in this subset had markers of more severe tremor, such as significantly higher TETRAS total scores and TETRAS performance subscores compared to isolated ET patients. Furthermore, ET plus patients had a significantly higher prevalence of head tremor compared to those with isolated ET, which may indicate more severe tremor or a separate ET subtype. Another study similarly showed more severe upper limb tremors and a higher prevalence of head tremor in ET plus patients [28]. Our ET plus patients also required treatment with significantly more medications than isolated ET patients. ET plus patients also had a significantly higher prevalence of dystonia than ET-PD patients (42.5% vs 9.0%, p < 0.0001).

A notable proportion of our patients (41.0%) received BoNT injections as part of their therapy, with at least partial improvement noted in 74.0%. This is similar to response rates in previous studies involving our center [29,30]. BoNT injections were similarly effective in all three cohorts, but were performed in significantly less patients with ET-PD.

Rest tremor has been increasingly recognized in ET, and indeed is one of the features included in the definition of ET plus [1]. Rest tremor was present in 66 of 72 (84.6%) ET-PD patients and 64 of 160 (40.0%) ET plus patients. There was an overall high prevalence of parkinsonism, with 26.0% of all patients classified as ET-PD and 13.0% of all patients classified as ET plus with parkinsonism. The mean latency between onset of tremor and onset of parkinsonism in our ET-PD patients was 21.0 years, suggesting PD with “antecedent ET” [1]. Another study examining ET-PD also noted a prolonged mean latency of onset of parkinsonism of 14 years [31].

Ataxic features, particularly balance disturbances and tandem gait abnormalities, have been frequently described in ET patients [32]. In one study, tandem gait was abnormal in 50% of 36 ET patients as compared to 28% of age-matched controls [33]. Several studies have provided evidence for a link between ET and cerebellar dysfunction and pathology [34,35,36]. In our study, subjective imbalance by history was present in 27.3% of all patients, and 23.0% had abnormal tandem gait on examination. Tandem gait abnormalities were significantly more prevalent among ET plus versus ET-PD patients (38.8% vs 9.0%, p < 0.0001). Other ataxic features were present in only 2.7% of all patients, suggesting mechanisms other than cerebellar degeneration accounting for the perception of problems with balance difficulties with tandem gait.

Non-motor features, particularly depression and anxiety, have been increasingly reported in patients with isolated ET [3,37,38,39,40,41,42,43]. However, other studies have not found a significant association between depression and ET [44]. Depression was reported in 29.7% and anxiety in 20.3% of all of our patients. While prevalence of depression was similar among the three cohorts, anxiety was significantly more prevalent in ET-PD versus isolated ET patients (30.8% vs 8.1%, p = 0.001).

Cognitive impairment as determined by MoCA screening or neuropsychological testing was present in 15.0% of all our patients, and there was no significant difference in prevalence between ET plus and ET-PD patients (16.3% vs 24.4%, p = 0.134). As expected and as noted in prior studies [45,46,47], patients with age at onset of tremor over 65 years had a significantly higher prevalence of cognitive impairment (37.0% vs 13.1%, p = 0.003). Several studies have shown cognitive deficits in ET patients exceeding those of age-matched [3,48,49] and historical controls [50]. A Spanish study found a higher incidence of dementia in ET patients versus controls (7.8% vs 3.9%, unadjusted relative risk = 2.08, p = 0.006) over a mean follow-up of 3.2 years, and ET patients over 65 were twice as likely to develop dementia compared to controls [46]. However, other studies have found variable frequency of cognitive deficit. One study of 83 elderly ET patients and 424 controls that showed no overall increase in the risk of dementia [51].

There has been an increasing interest in a relationship between ET and RBD and some studies have found the prevalence of RBD by screening questionnaires to be as high as 26.4% [52,53,54]. This is particularly relevant since RBD is an important risk factor for PD and, although still somewhat controversial, several studies have provided evidence that ET is a risk factor for PD [2,55,56,57]. Interestingly, in one study 100% of all patients with ET-RBD combination had rest tremor, compared with only 34.2% in the group with ET without RBD (p = 0.009) [52]. A much higher prevalence of RBD among ET-PD patients versus ET patients (51.9% vs 10%) was also noted in another study [58]. This is consistent with our study in which the prevalence of RBD was significantly higher among ET-PD patients compared to both isolated ET (15.4% vs 1.6%, p = 0.005) and ET plus patients (15.4% vs 2.5%, p = 0.0002). However, one study found no difference in self-reported RBD symptoms between ET-PD and ET plus patients with parkinsonism [59].

Our study has several limitations, most important of which is its retrospective nature. Retrospective data may often be difficult to fit into more current definitions. Some age at onset numbers required estimation from phrases used in chart documentation. Data on clinical features is only available when documented; lack of documentation may not necessarily mean that they were not present. On the other hand, retrospective data provides a more longitudinal and “real-world” perspective than prospective studies. Quantitative measures of tremor, parkinsonian features, mood, and cognitive function were not available in all patients. Assessment of treatment response was difficult because of lack of rating scales or other objective measures of response. Some information such as the number of return visits or reason for referral was not collected. Finally, the data is drawn entirely from a single tertiary referral center and, therefore, the findings may not be generalizable.

Reclassification of patients to ET plus potentially presents both clinical and research challenges [11,60]. When discussing the diagnosis of ET with patients it is important to communicate to them that many patients with ET may initially present with isolated tremor but additional features may emerge during the course of the disease. Further research is needed to determine whether isolated ET and ET plus represent a continuum of the same disorder or different subtypes with unique etiopathogenic mechanisms.

## Conclusion

While ET has been considered by some as an isolated tremor syndrome, it has been increasingly recognized to be accompanied by other motor features, such as parkinsonism, dystonia, and ataxia, and possibly non-motor features, including mood disturbances, cognitive impairment, sleep disorders, and hearing loss. The addition of “ET plus” as a diagnostic category in the Movement Disorder Society Consensus Statement [1] draws attention to these additional features. Since the publication, our and other studies [9,10,11,18] have found that most patients previously diagnosed as ET must be reclassified as ET plus. Furthermore, the additional, non-tremor features suggest the possibility of etiopathogenic subtypes.
